# Effective Parameters for 1D Photonic Crystals with Isotropic and Anisotropic Magnetic Inclusions: Coherent Wave Homogenization Theory

**DOI:** 10.3390/ma13061475

**Published:** 2020-03-24

**Authors:** J. Flores Méndez, A. C. Piñón Reyes, M. Moreno Moreno, A. Morales-Sánchez, Gustavo M. Minquiz, R. C. Ambrosio Lázaro, H. Vázquez Leal, F. Candia García

**Affiliations:** 1 Departamento de Ingeniería, Benemérita Universidad Autónoma de Puebla-Ciudad Universitaria, Blvd. Valsequillo y Esquina, Av. San Claudio s/n, Col. San Manuel, C.P. 72570 Puebla, Pue, Mexico; acpr_2417@hotmail.com (A.C.P.R.); gminquiz@yahoo.com (G.M.M.); roberto.ambrosio@correo.buap.mx (R.C.A.L.); filinc@hotmail.com (F.C.G.); 2 Tecnológico Nacional de México/I.T. Puebla-División de Estudios de Posgrado e Investigación, Av. Tecnológico No. 420, Maravillas, C.P. 72220 Puebla, Pue, Mexico; 3 Instituto Nacional de Astrofísica, Óptica y Electrónica, Luis Enrique Erro No. 1, 72840 Sta. Ma. Tonantzintla, Pue, Mexico; mmoreno@inaoep.mx (M.M.M.); alfredom@inaoep.mx (A.M.-S.); 4 Facultad de Instrumentación Electrónica, Universidad Veracruzana, Cto. Gonzalo Aguirre Beltrán S/N, 91000 Xalapa, Veracruz, Mexico; hvazquez@uv.mx; 5 Consejo Veracruzano de Investigación Científica y Desarrollo Tecnológico (COVEICYDET), Av. Rafael Murillo Vidal No. 1735, Cuauhtémoc, 91069 Xalapa, Veracruz, Mexico

**Keywords:** photonic crystal, homogenization theory, effective parameters, metamaterial

## Abstract

A homogenization theory that can go beyond the regime of long wavelengths is proposed, namely, a theory that is still valid for vectors of waves near the edge of the first zone of Brillouin. In this paper, we consider that the displacement vector and the magnetic induction fields have averages in the volume of the cell associated with the values of the electric and magnetic fields in the edges of the cell, so they satisfy Maxwell’s equations. Applying Fourier formalism, explicit expressions were obtained for the case of a photonic crystal with arbitrary periodicity. In the case of one-dimensional (1D) photonic crystals, the expressions for the tensor of the effective bianisotropic response (effective permittivity, permeability and crossed magneto-electric tensors) are remarkably simplified. Specifically, the effective permittivity and permeability tensors are calculated for the case of 1D photonic crystals with isotropic and anisotropic magnetic inclusions. Through a numerical calculation, the dependence of these effective tensors upon the filling fraction of the magnetic inclusion is shown and analyzed. Our results show good correspondence with the approach solution of Rytov’s effective medium. The derived formulas can be very useful for the design of anisotropic systems with specific optical properties that exhibit metamaterial behavior.

## 1. Introduction

The concept of metamaterial was initially introduced to explain the striking physical properties of photonic crystals, composed of resonant elements or having a very large dielectric contrast. For example, recently in [1], the design of a bilayer device (1D photonic crystal) made of dielectric materials on a substrate was reported. This membrane-type device enhances the Faraday rotation in comparison with plasmonic or metal-dielectric periodic structures, as well as showing good efficiency as a waveguide for transverse magnetic and transverse electric polarizations. Staude and Schilling [2] reviewed the optical properties of metamaterial-inspired silicon nanostructures, to explain the resonant frequency dependence of the optical metamaterial parameters, e.g., three-dimensional metamaterial-inspired arrangements of silicon nanoparticles having Mie resonances at a low frequency and behaving as double-negative electromagnetic mediums with simultaneously negative effective dielectric permittivity and magnetic permeability. Moreover, there are photonic crystals made of materials that are stimuli-responsive (the materials, either soft or aggregate, respond to thermal, pH, chemical, mechanical, optical, electrical or magnetic stimuli). With the application of these external stimuli, the range of electromagnetic frequencies in which there are no allowed propagation modes for any direction of the crystal are principally controlled. This can be exploited for applications in liquid–crystalline structures, sensors, optical phenomena diffraction and emission of photoluminescent materials. However, in recent years, scientists have faced a new challenge in studying the optical properties of these stimuli-responsive artificial materials in the area of nanophotonics. One of the great advances in this area is the design and manufacture of stimuli-responsive optical nanostructures (see, e.g., [3] and references therein). Thus, the most recent reports on these extraordinary structures offer great challenges and research opportunities in both physics and materials engineering, ranging from manufacturing techniques to the development of approximations and useful theories for the calculation of their effective electromagnetic magnitudes that allow us to characterize the control of the propagation of electromagnetic waves in the medium.

Magneto-dielectric photonic crystals are an interesting kind of metamaterial because of their unusual optical anisotropy [4,5]. The homogenization theories suggested in [4] and [5] provide accurate calculations to determine the effective refractive index for dielectric and magneto-dielectric 2D photonic crystals, respectively. Nowadays, homogenization theories have been proposed in order to calculate the effective optical parameters of metallo-dielectric photonic crystals with magnetic components (see, for example, [6,7,8,9,10,11,12,13] and references therein). All these theories characterize the electromagnetic oscillations in bianisotropic media, defined by the effective permeability, permittivity, and crossed magneto-electric tensors. Unfortunately, although the expressions for the effective parameters, obtained by some of these theories [4,5,6,9,11,12], are valid for photonic crystals with any type of Bravais lattice, their application in the case of 3D periodicity turns out to be impractical.

The purpose of this work is to present a homogenization theory that allows us to calculate the effective electromagnetic response of magneto-dielectric photonic crystals valid beyond the regime of long wavelengths, i.e., for wave vectors near the edge of the first zone of Brillouin. Explicit expressions are obtained for the tensors of the effective bianisotropic responses in the case of a photonic crystal with arbitrary periodicity, which, in the case of 1D photonic crystals, are significantly simplified. In developing our theory, called Coherent Wave Homogenization theory, we consider that the displacement vector (D) and the magnetic induction (B) fields have averages in the volume of the cell associated with the values of the electric (E) and magnetic (H) fields in the edges of the cell, so that they satisfy Maxwell’s equations. Furthermore, to determine the effective optical properties, we will efficiently use the general formalism of homogenization theories (see [14] and its references), based on Fourier formalism, which provides explicit formulas to determining all the components of the bianisotropic response tensors. Unlike previous theories, we present an approach for accurate calculations of the effective tensors in terms of the parameters of permittivity and permeability of the inclusion in the unit cell. The numerical implementation of the formulas obtained will be applied to 1D magneto-dielectric photonic crystals to study how the effective response of permeability and permittivity tensors behave versus the filling fraction and with the variation in the material parameters of the isotropic or anisotropic magnetic inclusions.

## 2. Mathematical Formulation: Homogenization Theory of Coherent Wave

### 2.1. Finite Fourier Transform

In this section, we shall derive an expression for the effective tensors based on the principle of wavelengths comparable with the lattice period for characterizing the bulk optical response of a homogenized magneto-dielectric photonic crystal. The most general form for such a response is the so-called bianisotropic response, generally written in the *EH*- (or Tellegen) representation, using the permittivity $\overset{↔}{\varepsilon }$ and permeability $\overset{↔}{\mu }$ tensors and two crossed magneto-electric dyadics, $\overset{↔}{\zeta }$ and $\overset{↔}{\xi }$, which depend on the position ($\vec{r}$) in the photonic crystal. Such tensorial representation relates the electric E and magnetic H fields with the displacement vector D and the magnetic induction B:

(1) $$D\left(\vec{r}\right)=\overset{↔}{\varepsilon }\left(\vec{r}\right)\cdot E\left(\vec{r}\right)+\overset{↔}{\xi }\left(\vec{r}\right)\cdot H\left(\vec{r}\right),$$

(2) $$B\left(\vec{r}\right)=\overset{↔}{\zeta }\left(\vec{r}\right)\cdot E\left(\vec{r}\right)+\overset{↔}{\mu }\left(\vec{r}\right)\cdot H\left(\vec{r}\right).$$

The behavior of these fields is governed by Maxwell equations:

(3) $$𝛻\times H\left(\vec{r}\right)=-i\omega D\left(\vec{r}\right),$$

(4) $$𝛻\cdot D\left(\vec{r}\right)=0,$$

(5) $$𝛻\times E\left(\vec{r}\right)=-i\omega B\left(\vec{r}\right),$$

(6) $$𝛻\cdot B\left(\vec{r}\right)=0.$$

Now, with Equations (1) and (2), we rewrite the laws of Ampere–Maxwell (in Equations (3) and (4)) and Faraday in (Equations (5) and (6)) in matrix form:

(7) $$\left[\begin{array}{cc} \overset{↔}{0} & 𝛻\times \overset{↔}{I}\\ -𝛻\times \overset{↔}{I} & \overset{↔}{0} \end{array}\right]\cdot v\left(\vec{r}\right)=-i\omega w\left(\vec{r}\right).$$

In this latter equation, the dyadic unit and zero are introduced ($\overset{↔}{I}$ and $\overset{↔}{0}$, respectively), as well as the vectors *EH* and *DB* ($v\left(\vec{r}\right)$ and $w\left(\vec{r}\right)$, respectively), given by:

(8) $$v\left(\vec{r}\right)\equiv \left[\begin{array}{c} E\left(\vec{r}\right)\\ H\left(\vec{r}\right) \end{array}\right],\;w\left(\vec{r}\right)\equiv \left[\begin{array}{c} D\left(\vec{r}\right)\\ B\left(\vec{r}\right) \end{array}\right]=\overset{=}{A}\left(\vec{r}\right)v\left(\vec{r}\right)$$

where $\overset{=}{A}\left(\vec{r}\right)$ is a 6 × 6 matrix defined by:

(9) $$\overset{=}{A}\left(\vec{r}\right)\equiv \left[\begin{array}{cc} \overset{↔}{\varepsilon }\left(\vec{r}\right) & \overset{↔}{\xi }\left(\vec{r}\right)\\ \overset{↔}{\zeta }\left(\vec{r}\right) & \overset{↔}{\mu }\left(\vec{r}\right) \end{array}\right]$$

Due to the periodicity of the bianisotropic response tensors, we write the matrix $\overset{=}{A}\left(\vec{r}\right)$ in an expansion in Fourier series:

(10) $$\overset{=}{A}\left(\vec{r}\right)=\underset{\vec{G}}{\sum }\overset{=}{A}\left(\vec{G}\right)e^{i\vec{G}\cdot \vec{r}}$$

the summation extends over the vectors of the reciprocal lattice ($\vec{G}$) of the photonic crystal.

Moreover, we can expand the vectors in Bloch waves due to the periodicity along the plane parallel to one of the surfaces of the photonic crystal:

(11) $$v\left(\vec{r}\right)=\sum_{{\vec{G}}_{\left|\right|}}e^{i{\vec{q}}_{\left|\right|}\cdot {\vec{r}}_{\left|\right|}}v\left({\vec{G}}_{\left|\right|},z\right)e^{i{\vec{G}}_{\left|\right|}\cdot {\vec{r}}_{\left|\right|}},$$

where ${\vec{G}}_{\left|\right|}$ is a vector of the reciprocal lattice of the plane perpendicular to the *z*-direction.

Substituting Equation (11) into Equation (7), we get:

(12) $$\left[\begin{array}{cc} \overset{↔}{0} & \left(i\left({\vec{q}}_{\left|\right|}+{\vec{G}}_{\left|\right|}\right)+\hat{z}\frac{\partial }{\partial z}\right)\times \overset{↔}{I}\\ -\left(i\left({\vec{q}}_{\left|\right|}+{\vec{G}}_{\left|\right|}\right)+\hat{z}\frac{\partial }{\partial z}\right)\times \overset{↔}{I} & \overset{↔}{0} \end{array}\right]v\left({\vec{G}}_{\left|\right|},z\right)=-i\omega w\left({\vec{G}}_{\left|\right|},z\right)$$

Now, we will apply the finite Fourier transform in an interval along the *z*-axis of length equal to the lattice constant *a*:

(13) $$F\left(G_{z}\right)=\frac{1}{a}\int_{-a/2}^{a/2}f\left(z\right)e^{-iG_{z}z}dz,$$

where $G_{z}=\frac{2\pi n_{z}}{a}$, $n_{z}$=−∞, … , ∞. The inverse transform is given by the series:

(14) $$f\left(z\right)=\sum_{G_{z}}e^{iG_{z}z}f\left(G_{z}\right)\;,\;\mathrm{for}-\frac{a}{2}\leq z\leq \frac{a}{2}$$

From Equations (12) and (13), we obtain:

(15) $$\left[\begin{array}{cc} \overset{↔}{0} & \left(i\left({\vec{q}}_{\left|\right|}+{\vec{G}}_{\left|\right|}\right)+\hat{z}\frac{\partial }{\partial z}\right)\times \overset{↔}{I}\\ -\left(i\left({\vec{q}}_{\left|\right|}+{\vec{G}}_{\left|\right|}\right)+\hat{z}\frac{\partial }{\partial z}\right)\times \overset{↔}{I} & \overset{↔}{0} \end{array}\right]v\left({\vec{G}}_{\left|\right|},G_{z}\right)dz+\;\left[\begin{array}{cc} \overset{↔}{0} & \frac{\hat{z}}{a}\times \overset{↔}{I}\\ -\frac{\hat{z}}{a}\times \overset{↔}{I} & \overset{↔}{0} \end{array}\right]\left[v\left({\vec{G}}_{\left|\right|},z=a\right)-v\left({\vec{G}}_{\left|\right|},z=0\right)\right]=-i\omega w\left({\vec{G}}_{\left|\right|},G_{z}\right).$$

Rewriting this equation and considering that:

(16) $$w\left({\vec{G}}_{\left|\right|},G_{z}\right)=\sum_{\vec{G}\prime_{\left|\right|},G\prime_{z}}\overset{=}{A}\left({\vec{G}}_{\left|\right|}-\vec{G}\prime_{\left|\right|},G_{z}-G\prime_{z}\right)v\left(\vec{G}\prime_{\left|\right|},{G^{\prime }}_{z}\right),$$

here, $\overset{=}{A}$ is the matrix of the bianisotropic response. Therefore, Equation (15) can be rewritten as:

(17) $$\underset{\vec{G}\prime_{\left|\right|},{G^{\prime }}_{z}}{\sum }\overset{=}{D}\left({\vec{G}}_{\left|\right|},G_{z},\vec{G}\prime_{\left|\right|},{G^{\prime }}_{z}\right)v\left({\vec{G}}_{\left|\right|},G_{z}\right)=\left[\begin{array}{cc} \overset{↔}{0} & \frac{\hat{z}}{a}\times \overset{↔}{I}\\ -\frac{\hat{z}}{a}\times \overset{↔}{I} & \overset{↔}{0} \end{array}\right]\left[v\left({\vec{G}}_{\left|\right|},z=a\right)-v\left({\vec{G}}_{\left|\right|},z=0\right)\right]$$

where:

(18) $$\overset{=}{D}\left({\vec{G}}_{\left|\right|},G_{z},\vec{G}\prime_{\left|\right|},{G^{\prime }}_{z}\right)=\left[\begin{array}{cc} \overset{↔}{0} & \left(i\left({\vec{q}}_{\left|\right|}+{\vec{G}}_{\left|\right|}\right)+i\hat{z}G_{z}\right)\times \overset{↔}{I}\\ -\left(i\left({\vec{q}}_{\left|\right|}+{\vec{G}}_{\left|\right|}\right)+i\hat{z}G_{z}\right)\times \overset{↔}{I} & \overset{↔}{0} \end{array}\right]\delta_{{\vec{G}}_{\left|\right|},G_{z};\vec{G}\prime_{\left|\right|},{G^{\prime }}_{z}}+\;i\omega \overset{=}{A}\left({\vec{G}}_{\left|\right|}-\vec{G}\prime_{\left|\right|},G_{z}-G\prime_{z}\right)$$

The amplitudes $v\left({\vec{G}}_{\left|\right|},z=0\right)$ and $v\left({\vec{G}}_{\left|\right|},z=a\right)$ in Equation (17) are defined by the expressions:

(19) $$v\left({\vec{G}}_{\left|\right|},z=0\right)=\sum_{G_{z}}v\left({\vec{G}}_{\left|\right|},G_{z}\right)e^{iG_{z}0^{+}}$$

(20) $$v\left({\vec{G}}_{\left|\right|},z=a\right)=\underset{G_{z}}{\sum }v\left({\vec{G}}_{\left|\right|},G_{z}\right)e^{iG_{z}a^{-}}$$

Using Equations (17)–(19), a homogeneous system is obtained for the amplitudes $v\left({\vec{G}}_{\left|\right|},z=0\right)$ and $v\left({\vec{G}}_{\left|\right|},z=a\right)$. Therefore, the amplitudes for ${\vec{G}}_{\left|\right|}\neq 0$ can be expressed in terms of one of them, for example, $v\left({\vec{G}}_{\left|\right|}=0,z=0\right)$.

Now, let us determine the effective response. From Equations (17) and (19), we have:

(21) $$v\left({\vec{G}}_{\left|\right|},z=0\right)=-\sum_{G_{z},\vec{G}\prime_{\left|\right|},{G^{\prime }}_{z}}{\overset{=}{D}}^{-1}\left({\vec{G}}_{\left|\right|},G_{z},\vec{G}\prime_{\left|\right|},{G^{\prime }}_{z}\right)e^{iG_{z}0^{+}}\left[\begin{array}{cc} \overset{↔}{0} & \frac{\hat{z}}{a}\times \overset{↔}{I}\\ -\frac{\hat{z}}{a}\times \overset{↔}{I} & \overset{↔}{0} \end{array}\right]\times \;\left[v\left(\vec{G}\prime_{\left|\right|},z=a\right)-v\left(\vec{G}\prime_{\left|\right|},z=0\right)\right]$$

This expression can be written as:

(22) $$\left[\begin{array}{cc} \overset{↔}{0} & \frac{\hat{z}}{a}\times \overset{↔}{I}\\ -\frac{\hat{z}}{a}\times \overset{↔}{I} & \overset{↔}{0} \end{array}\right]\left[v\left({\vec{G}}_{\left|\right|},z=a\right)-v\left({\vec{G}}_{\left|\right|},z=0\right)\right]=\;-\sum_{\vec{G}\prime_{\left|\right|}}{\left\{\sum_{G_{z},G\prime_{z}}{\overset{=}{D}}^{-1}\left({\vec{G}}_{\left|\right|},G_{z},\vec{G}\prime_{\left|\right|},{G^{\prime }}_{z}\right)e^{iG_{z}0^{+}}\right\}}^{-1}v\left(\vec{G}\prime_{\left|\right|},z=0\right).$$

Evaluating this equation in ${\vec{G}}_{\left|\right|}=0$, we obtain an equation for the coherent component:

(23) $$\left[\begin{array}{cc} \overset{↔}{0} & \frac{\hat{z}}{a}\times \overset{↔}{I}\\ -\frac{\hat{z}}{a}\times \overset{↔}{I} & \overset{↔}{0} \end{array}\right]\left[v\left({\vec{G}}_{\left|\right|}=0,z=a\right)-v\left({\vec{G}}_{\left|\right|}=0,z=0\right)\right]=-\sum_{\vec{G}\prime_{\left|\right|}}{\left\{\sum_{G_{z},G\prime_{z}}{\overset{=}{D}}^{-1}\left({\vec{G}}_{\left|\right|}=\;0,G_{z},\vec{G}\prime_{\left|\right|},{G^{\prime }}_{z}\right)e^{iG_{z}0^{+}}\right\}}^{-1}v\left(\vec{G}\prime_{\left|\right|},z=0\right).$$

In the bulk of the photonic crystal, the Bloch theorem must be satisfied, $v\left({\vec{G}}_{\left|\right|}=0,z=a\right)=e^{iq_{z}a}v\left({\vec{G}}_{\left|\right|}=0,z=0\right)$, so the term on the left side in Equation (23) can be written as:

(24) $$\frac{1}{a}\left[v\left({\vec{G}}_{\left|\right|}=0,z=a\right)-v\left({\vec{G}}_{\left|\right|}=0,z=0\right)\right]=\langle \frac{d}{dz}e^{iq_{z}a}\rangle v\left({\vec{G}}_{\left|\right|}=0,z=0\right)\;=iq_{z}\langle e^{iq_{z}a}\rangle v\left({\vec{G}}_{\left|\right|}=0,z=0\right)$$

Substituting this into Equation (23), we arrive at the equations for the coherent amplitude:

(25) $$\left[\begin{array}{cc} \overset{↔}{0} & iq_{z}\hat{z}\times \overset{↔}{I}\\ -iq_{z}\hat{z}\times \overset{↔}{I} & \overset{↔}{0} \end{array}\right]v\left({\vec{G}}_{\left|\right|}=0,z=0\right)\;=-\frac{1}{\left\langle e^{iq_{z}a}\right\rangle }\cdot \underset{\vec{G}\prime_{\left|\right|}}{\sum }{\left\{\underset{G_{z},G\prime_{z}}{\sum }{\overset{=}{D}}^{-1}\left({\vec{G}}_{\left|\right|}=0,G_{z},\vec{G}\prime_{\left|\right|},{G^{\prime }}_{z}\right)e^{iG_{z}0^{+}}\right\}}^{-1}v\left(\vec{G}\prime_{\left|\right|},z=0\right)$$

which correspond to the Maxwell equations for the amplitude of the field *E*–*H*, propagating in the *z*-direction, in a homogeneous medium:

(26) $$\left[\begin{array}{cc} \overset{↔}{0} & iq_{z}\hat{z}\times \overset{↔}{I}\\ -iq_{z}\hat{z}\times \overset{↔}{I} & \overset{↔}{0} \end{array}\right]v\left({\vec{G}}_{\left|\right|}=0,z=0\right)=-i\omega w_{eff},$$

where

(27) $$w_{eff}={\overset{=}{A}}_{eff}v\left({\vec{G}}_{\left|\right|}=0,z=0\right),$$

is the effective field *D*–*B*. The effective matrix $\overset{=}{A}$ of the bianisotropic response is obtained directly from Equations (25) and (27), and the relationship between the coefficients $v\left(\vec{G}\prime_{\left|\right|},z=0\right)$ and the coherent amplitude $v\left({\vec{G}}_{\left|\right|}=0,z=0\right)$ mentioned above (Equation (21)). Therefore, we have explicit expressions to calculate the effective tensors of the bianisotropic response of a homogenized photonic crystal without any restrictions on the wave vector $q_{z}$.

### 2.2. 1D Photonic Crystals

Let us calculate the effective parameters for a homogenized binary (bilayer) 1D photonic crystal. The Fourier coefficients $\overset{=}{A}\left(\vec{G}\right)$ in Equation (16) are given by Equation (28); the crossed magnetoelectric tensors are assumed to be equal to 3 × 3 zero matrices:

(28) $$\overset{=}{A}\left(G_{z}-{G^{\prime }}_{z}\right)=\left[\begin{array}{cc} \overset{↔}{I}\varepsilon \left(G_{z}-{G^{\prime }}_{z}\right) & \overset{↔}{0}\\ \overset{↔}{0} & \overset{↔}{I}\mu \left(G_{z}-{G^{\prime }}_{z}\right) \end{array}\right]$$

where

(29) $$\varepsilon \left(G_{z}-{G^{\prime }}_{z}\right)=\frac{1}{a}\int_{0}^{a}\varepsilon \left(z\right)e^{-i\left(G_{z}-{G^{\prime }}_{z}\right)z}d_{z},$$

effecting the integral on the unit cell, the following expression is obtained:

(30) $$\varepsilon \left(G_{z}-{G^{\prime }}_{z}\right)=e^{-i\left(G_{z}-{G^{\prime }}_{z}\right)a/2}\left[\varepsilon_{b}\delta_{G_{z},{G^{\prime }}_{z}}+∆\varepsilon \;F\left(G_{z}-{G^{\prime }}_{z}\right)\right].$$

The matrix $\overset{=}{D}$ (Equation (18)), acquires the simplest form:

(31) $$\overset{=}{D}\left(G_{z},G\prime_{z}\right)=\left[\begin{array}{cc} \overset{↔}{0} & \left({\vec{q}}_{\left|\right|}+\hat{z}G_{z}\right)\times \overset{↔}{I}\\ -\left({\vec{q}}_{\left|\right|}+\hat{z}G_{z}\right)\times \overset{↔}{I} & \overset{↔}{0} \end{array}\right]\delta_{G_{z},G\prime_{z}}+\omega \overset{=}{A}\left(G_{z}-G\prime_{z}\right),\;$$

and it satisfies

(32) $$\sum_{G\prime_{z}}\overset{=}{D}\left(G_{z},G\prime_{z}\right){\overset{=}{D}}^{-1}\left(G\prime_{z},G{\prime \prime }_{z}\right)=\overset{↔}{I}\;\delta_{G_{z},G{\prime \prime }_{z}}.$$

On the other hand, the effective matrix takes the form:

(33) $${\overset{=}{A}}_{eff}=\frac{1}{\omega }\frac{1}{\left\langle e^{iq_{z}z}\right\rangle }{\left\{\underset{G_{z},G\prime_{z}}{\sum }{\overset{=}{D}}^{-1}\left(G_{z},G\prime_{z}\right)e^{iG_{z}0^{+}}\right\}}^{-1}$$

Let us introduce

(34) $${\tilde{D}}^{-1}\left(G\prime_{z}\right)=\underset{G_{z}}{\sum }e^{iG_{z}0^{+}}\;{\overset{=}{D}}^{-1}\left(G_{z},G\prime_{z}\right)$$

so, the effective matrix (Equation (33)) acquires the simplest form:

(35) $${\overset{=}{A}}_{eff}=\frac{1}{\omega }\frac{1}{\left\langle e^{iq_{z}z}\right\rangle }{\left\{\underset{G\prime_{z}}{\sum }{\tilde{D}}^{-1}\left(G\prime_{z}\right)\right\}}^{-1}$$

Note that this expression depends on $q_{z}$, which in general is non-local, i.e., the effective parameters depend on both frequency *ω* and the wave vector $q_{z}$.

## 3. Implementation and Results

**Figure 1:**
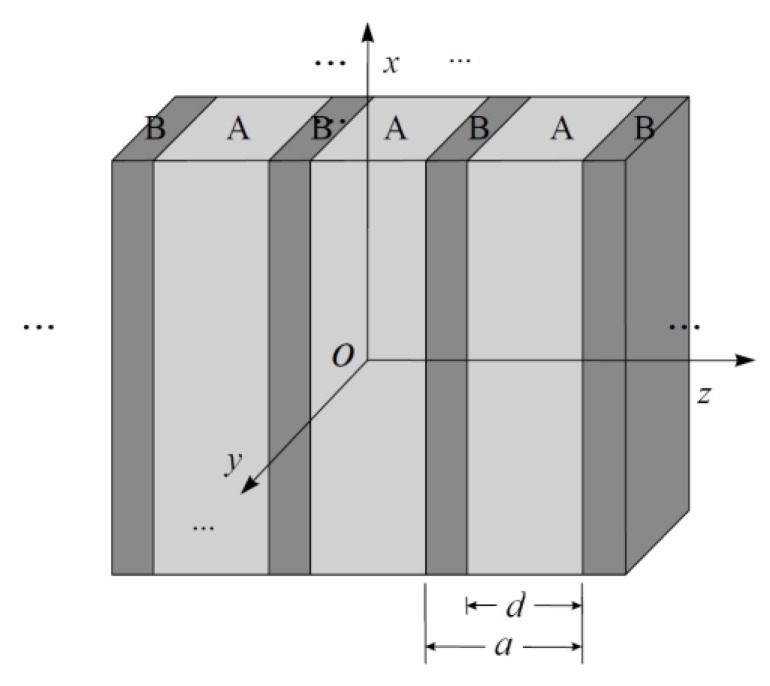
One-dimensional (1D) photonic crystal or bilayer superlattice with periodicity in *z*-direction.

In this section, we apply the Coherent Wave Homogenization theory developed in the previous section for calculating the effective permeability and permittivity tensors versus the filling-fraction of 1D magneto-dielectric photonic crystals. Here, it is convenient to indicate that in the case of alternating layers being sufficiently thin in comparison with the characteristic wavelength, we can treat the whole system as an anisotropic medium with effective response tensors (we consider that the Bloch wavelength is much greater than the lattice parameter of the photonic crystal, $q_{z}$*a* << 1). As follows from Figure 1 and according to the previous conditions for wavelength frequencies in THz, a lattice parameter *a* = 0.15 µm is proposed, *d* is the thickness of the inclusion layer and for the case of a superlattice with two alternating layers, the filling fraction f=d/a, and is valid for values of *d* in the range *0 ≤ d ≤ a*. In this work, the interest in the THz regime becomes more attractive due to the huge technological applications, e.g., telecommunications, sensing and astronomy. In general, the periodicity of the photonic structure will be proportional to the wavelength of the electromagnetic radiation to be controlled. Therefore, for homogenized photonic crystals of arbitrary thickness, the effective electromagnetic magnitudes of magnetic permeability and dielectric permittivity that characterize the propagation of electromagnetic waves in the medium can always be determined. Here, we will study photonic crystals whose bilayer unitary cell is composed of a layer of dielectric material with inclusions of magnetic–isotropic and magnetic–anisotropic media. The constituents of the unit cell will be considered in a usual manner—that is, medium “A” will correspond to the inclusion and medium “B” to the background or host (see Figure 1).

### 3.1. 1D Photonic Crystals with Isotropic Inclusion

**Figure 2:**
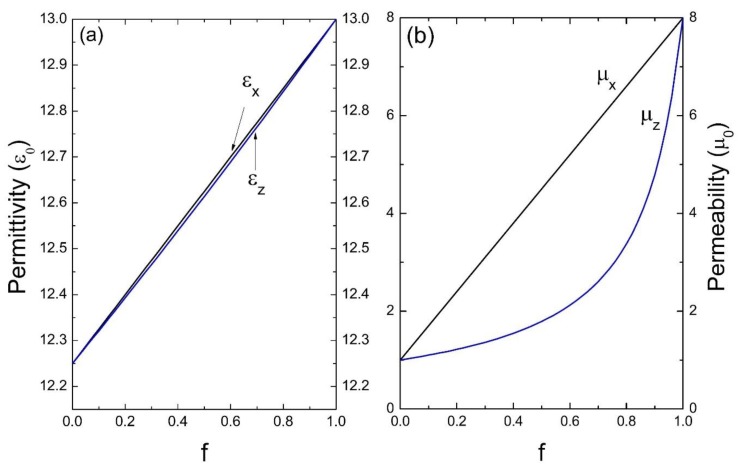
Permittivity ε (**a**) and permeability μ (**b**) versus the filling fraction *f* (ε_0_ and μ_0_ are the units).

**Figure 3:**
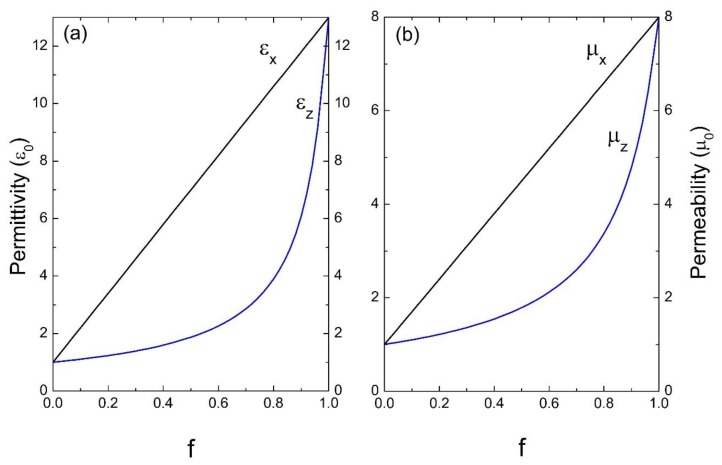
Permittivity ε (**a**) and permeability μ (**b**) versus the filling fraction *f* (ε_0_ and μ_0_ are the units).

Now, using Equation (35), we have numerically calculated the 6 × 6 matrix ${\overset{=}{A}}_{eff}$ of the effective tensor of the bianisotropic response, in the local limit ($q_{z}$→0) with a total number of *n_z_ =* 101 waves. As an initial application, we consider photonic crystals composed of a homogeneous host and isotropic inclusions. Specifically, in Figure 2, we present the results for a superlattice whose unit cell is composed of a magnetic material, in this case isotropic ferrite, considered as medium “A”, and a dielectric material homogeneous to silicon, as medium “B”; the parameters considered for this calculation were: ε_A_ = 13ε_0_, μ_A_ = 8μ_0_ and ε_B_ = 12.25ε_0_, μ_B_ = μ_0_, respectively (ε_0_ and μ_0_ are the permittivity and permeability of the vacuum, respectively). In this figure we show the permittivity and the effective permeability as a function of the filling fraction *f*, note that ε_x_ = ε_y_ and μ_x_ = μ_y_, since there is isotropy in the *x* − *y* plane. Figure 3 corresponds to a unit cell formed by ferrite in air; the parameters considered were: ε_A_ = 13ε_0_, μ_A_ = 8μ_0_ and ε_B_ = ε_0_, μ_B_ = μ_0_.

**Figure 4:**
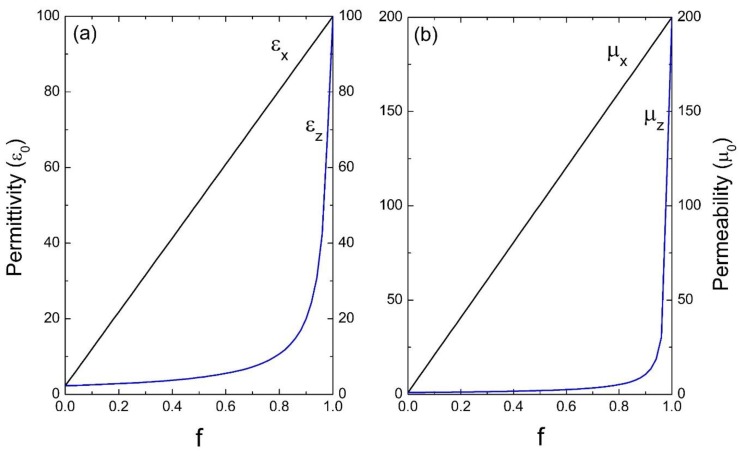
Permittivity ε (**a**) and permeability μ (**b**) versus the filling fraction *f* (ε_0_ and μ_0_ are the units).

**Figure 5:**
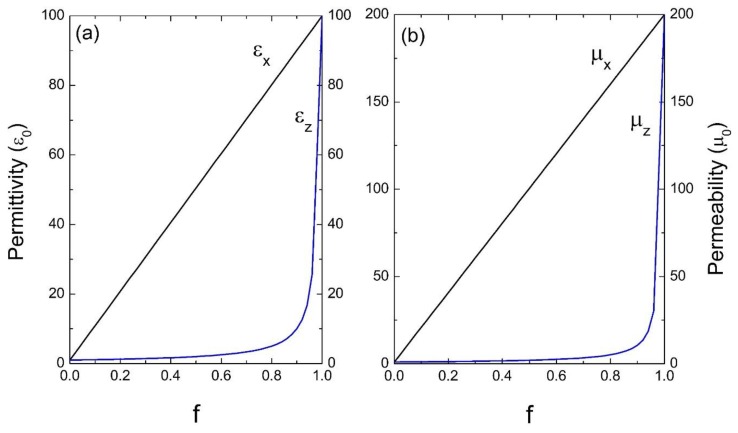
Permittivity ε (**a**) and permeability μ (**b**) versus the filling fraction *f* (ε_0_ and μ_0_ are the units).

Next, we considered photonic crystals with high dielectric and magnetic contrast in their unit cell. Figure 4 corresponds to a system of common glass in a homogeneous dielectric as the host, the parameters used were: ε_A_ = 100ε_0_, μ_A_ = 200μ_0_ and ε_B_ = 2.25ε_0_, μ_B_ = μ_0_. Finally, in Figure 5, a medium of common glass in air is considered, whose parameters are: ε_A_ = 100ε_0_, μ_A_ = 200μ_0_ and ε_B_ = ε_0_, μ_B_ = μ_0_.

Figure 2, Figure 3, Figure 4 and Figure 5 indicate the averages of the permittivity and the permeability in the unit cell and it is observed that the increase in the values of ε_x_ (=ε_y_) and μ_x_ (=μ_y_), as well as in ε_z_ and μ_z_ in the function of the filling fraction present a linear and non-linear behavior, respectively. Therefore, our results describe the effective medium approach proposed by Rytov [15]—if the materials of the superlattice layers are local and isotropic, the permittivity tensor is diagonal with principal values [15]:

(36) $$\varepsilon_{x}=\varepsilon_{y}=\varepsilon_{m}f+\varepsilon_{d}\left(1-f\right),$$

(37) $$\frac{1}{\varepsilon_{z}}=\frac{f}{\varepsilon_{m}}=\frac{1-f}{\varepsilon_{d}}.\;$$

Here, the metal and dielectric layers are characterized by their permittivity, εm and εd; f is the metal filling fraction and z denotes the direction perpendicular to the planes of the layers in the superlattice. Equations similar to the previous ones are fulfilled for the magnetic media:

(38) $$\mu_{x}=\mu_{y}=\mu_{m}f+\mu_{d}\left(1-f\right)$$

(39) $$\frac{1}{\mu_{z}}=\frac{f}{\mu_{m}}=\frac{1-f}{\mu_{d}}.$$

Evidently, when f →0, the medium “B” predominates and as the filling fraction increases, namely f →1, the material will correspond to the inclusion. On the other hand, in Figure 4 and Figure 5 it is observed how for the effective electric permittivity and the effective magnetic permeabilities in the z-component for filling fractions near 0.8, their values remain very close to the host, later ascending rapidly before reaching the value of inclusion. This is due to the high contrast dielectric and magnetic qualities of the materials in the unit cell, the contrast being defined as the ratio of medium “A” and medium “B”—that is, εA/εB and μA/μB, respectively. Note that this behavior is not presented in Figure 2 and Figure 3 due to the low dielectric and magnetic contrast between the host and the inclusion.

### 3.2. 1D Photonic Crystals with Anisotropic Inclusion

In the applications that follow, we calculate the effective parameters of a photonic crystal whose unit cell is formed by an anisotropic inclusion and isotropic background in the dielectric constant and magnetic permeability.

**Figure 6:**
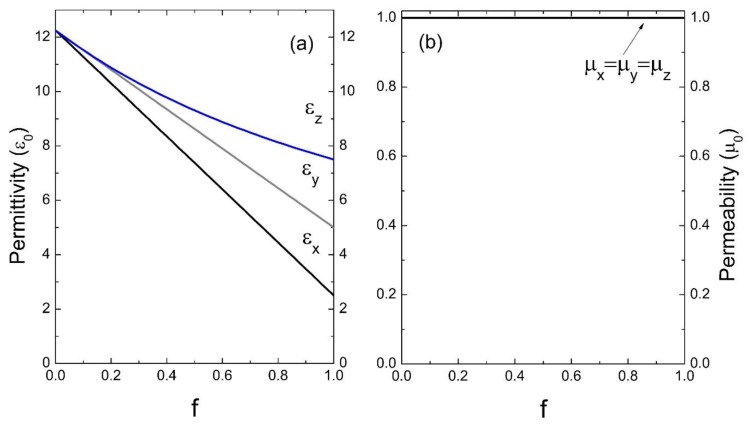
Permittivity ε (**a**) and permeability μ (**b**) versus the filling fraction *f* (ε_0_ and μ_0_ are the units). The parameters used were: ε_Ax_ = 2.5ε_0_, ε_Ay_ = 5ε_0_, ε_Az_ = 7.5ε_0_, μ_A_ = μ_0_ (anisotropic media) and ε_B_ = 12.25ε_0_, μ_B_ = μ_0_ (isotropic media).

Figure 6a shows the results for a superlattice where the inclusion presents anisotropy in the dielectric constant; as can be observed, as the amount of the anisotropic medium increases (medium "A"), the effective response becomes anisotropic, creating three well-defined values—that is, when the filling fraction tends towards zero (*f* →0), the system is an isotropic homogenous medium with permittivity ε_B_. In the opposite case, when *f* →1 the system is an anisotropic homogenous medium with permittivity ε_A_. Note that ε_x_ and ε_y_ maintain a linear behavior determined by Equation (36), while the component ε_z_ is represented by a curve defined by the result of the inverse relationship of Equation (37). In the case of effective permeability (see Figure 6b), a linear behavior is observed, as determined by Equation (38), which indicates an isotropic behavior for any filling fraction of the inclusion. Moreover, from the inspection of Figure 6b, it is evident that μ_x_ = μ_y_ = μ_z_, considering that both the inclusion and the host have a relative permeability approximately equal to one (vacuum permeability).

**Figure 7:**
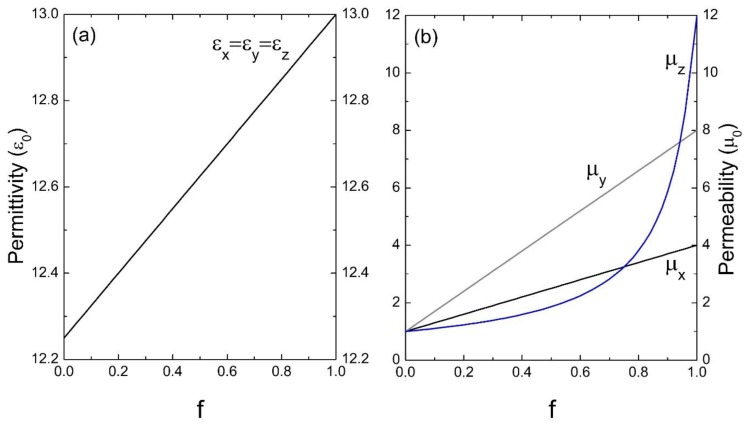
Permittivity ε (**a**) and permeability μ (**b**) versus the filling fraction *f* (ε_0_ and μ_0_ are the units). The parameters used were: ε_A_ = 13ε_0_, μ_Ax_ = 4μ_0_, μ_Ay_ = 8μ_0_, μ_Az_ = 12μ_0_ and ε_B_ = 12.25ε_0_, μ_B_ = μ_0_.

On the other hand, the effective parameters of a superlattice whose inclusion presents anisotropy in the magnetic permeability are determined. Therefore, the effective permittivity turns out to be isotropic throughout the filling fraction of the inclusion (see Figure 7a) and shows a linear behavior (ε_x_ = ε_y_ = ε_z_), as determined in the first approximation by Equation (36). However, in Figure 7b, the situation is somewhat different because as the amount of the anisotropic medium increases, the effective response becomes anisotropic—that is, when *f* →0 the homogeneous medium is isotropic and when *f* →1 the homogeneous medium is clearly anisotropic. However, it should be noted that μ_x_ and μ_y_ maintain a linear behavior determined by Equation (38), while the μ_z_ component is represented by a curve which is defined by Equation (39).

**Figure 8:**
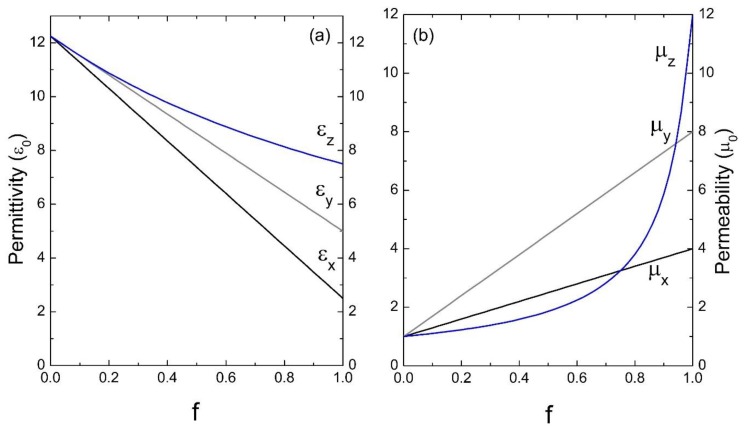
Permittivity ε (**a**) and permeability μ (**b**) versus the filling fraction *f* (ε_0_ and μ_0_ are the units). The parameters used were: ε_Ax_ = 2.5ε_0_, ε_Ay_ = 5ε_0_, ε_Az_ = 7.5ε_0_, μ_Ax_ = 4μ_0_, μ_Ay_ = 8μ_0_, μ_Az_ = 12μ_0_ and ε_B_ = 12.25ε_0_, μ_B_ = μ_0_.

Finally, Figure 8 shows the results of the effective response for a photonic crystal whose inclusion in its unit cell presents anisotropy in the dielectric permittivity and magnetic permeability. As can be seen in both graphs, the physical situation is similar to that already discussed in Figure 6a and Figure 7b for the effective permittivity and permeability, respectively, where it is basically noted that when increasing the filling fraction of the anisotropic medium, the effective response is anisotropic.

## 4. Conclusions

In this work, a homogenization theory for 1D magneto-dielectric photonic crystals was presented. The theory developed reports of analytical expressions that allow us to calculate the effective components of the permittivity and permeability tensors. In particular, we have analyzed the case of photonic crystals whose bilayer unitary cell is composed of a layer of dielectric material with inclusions of magnetic–isotropic and magnetic–anisotropic media, in the local limit, when the wavelength of the work frequency of the incident radiation on the medium is large compared to the period of the unit cell. The numerical implementation of the formulas provides results for new types of homogenized photonic metamaterials; in particular, we have studied theoretically the anisotropy of effective magnetic permeability and effective dielectric permittivity for homogenized magneto-dielectric binary superlattices versus the filling fraction. We demonstrated that the principal values for the components of the permeability and permittivity effective tensors describe a regime where Rytov’s formulas are valid. Our results may be useful for the better comprehension and design of metamaterials, due to the anisotropy that they present in electro-magnetic modes, which can be manufactured even with structures as simple superlattices.
