# Erratum: Flores, J., et al. Effective Parameters for 1D Photonic Crystals with Isotropic and Anisotropic Magnetic Inclusions: Coherent Wave Homogenization Theory. *Materials* 2020, *13*, 1475

**DOI:** 10.3390/ma13143053

**Published:** 2020-07-08

**Authors:** J. Flores Méndez, A. C. Piñón Reyes, M. Moreno Moreno, A. Morales-Sánchez, Gustavo M. Minquiz, R. C. Ambrosio Lázaro, H. Vázquez Leal, F. Candia García

**Affiliations:** 1 Departamento de Ingeniería, Benemérita Universidad Autónoma de Puebla-Ciudad Universitaria, Blvd. Valsequillo y Esquina, Av. San Claudio s/n, Col. San Manuel, C.P. 72570 Puebla, Pue, Mexico; acpr_2417@hotmail.com (A.C.P.R.); gminquiz@yahoo.com (G.M.M.); roberto.ambrosio@correo.buap.mx (R.C.A.L.); filinc@hotmail.com (F.C.G.); 2 Tecnológico Nacional de México/I.T. Puebla-División de Estudios de Posgrado e Investigación, Av. Tecnológico No. 420, Maravillas, C.P. 72220 Puebla, Pue, Mexico; 3 Instituto Nacional de Astrofísica, Óptica y Electrónica, Luis Enrique Erro No. 1, 72840 Sta. Ma. Tonantzintla, Pue, Mexico; mmoreno@inaoep.mx (M.M.M.); alfredom@inaoep.mx (A.M.-S.); 4 Facultad de Instrumentación Electrónica, Universidad Veracruzana, Cto. Gonzalo Aguirre Beltrán S/N, 91000 Xalapa, Veracruz, Mexico; hvazquez@uv.mx; 5 Consejo Veracruzano de Investigación Científica y Desarrollo Tecnológico (COVEICYDET), Av. Rafael Murillo Vidal No. 1735, Cuauhtémoc, 91069 Xalapa, Veracruz, Mexico

The authors wish to make the following corrections to this paper [1]: replace:

(37) $$\frac{1}{\varepsilon_{z}}=\frac{f}{\varepsilon_{m}}=\frac{1-f}{\varepsilon_{d}}$$

and

(39) $$\frac{1}{\mu_{z}}=\frac{f}{\mu_{m}}=\frac{1-f}{\mu_{d}}$$

with the correct expressions:

(37) $$\frac{1}{\varepsilon_{z}}=\frac{f}{\varepsilon_{m}}+\frac{1-f}{\varepsilon_{d}}$$

and

(39) $$\frac{1}{\mu_{z}}=\frac{f}{\mu_{m}}+\frac{1-f}{\mu_{d}}$$

These changes have no material impact on the conclusions of the paper.

The authors would like to apologize for any inconvenience caused to the readers by these changes.
